# Genome-wide (ChIP-seq) identification of target genes regulated by WRKY33 during submergence stress in *Arabidopsis*

**DOI:** 10.1186/s12863-021-00972-5

**Published:** 2021-05-24

**Authors:** Junlin Zhang, Bao Liu, Yan Song, Yang Chen, Jiao Fu, Jianquan Liu, Tao Ma, Zhenxiang Xi, Huanhuan Liu

**Affiliations:** Key Laboratory for Bio-resources and Eco-environment & State Key Lab of Hydraulics & Mountain River Engineering, College of Life Science, Sichuan University, Chengdu, 610065 China

**Keywords:** *WRKY33*, Submergence treatment, Hypoxia, ChIP-seq, *Arabidopsis*

## Abstract

**Background:**

Hypoxia induced by flooding causes significant losses to crop production almost every year. However, the molecular network of submergence signaling pathway is still poorly understood. According to previous studies, transgenic plants overexpressing the *WRKY33* gene showed enhanced resistance to submergence stress. Thus, this transcription factor may regulate a series of target genes in response to submergence. Here, to determine putative downstream targets of WRKY33 at a genome-wide scale in *Arabidopsis thaliana*, we performed the chromatin immunoprecipitation sequencing (ChIP-seq) using *35S:FLAG-WRKY33* overexpression transgenic lines (*WRKY33-OE*) after 24 h of submergence treatment.

**Results:**

Using ChIP-seq data, we identified a total of 104 WRKY33-binding genes under submergence stress (WRKY33BGSs). Most WRKY33BGSs are involved in the oxidation-reduction process, programmed cell death in response to reactive oxygen species, lipid biosynthesis process, and other processes related to stress responses. Moreover, the major motif identified in the WRKY33BGSs promoters is a new *cis*-element, TCTCTC (named here as “TC box”). This *cis*-element differs from the previously known W box for WRKY33. Further qPCR experiments verified that genes carrying this motif in their promoters could be regulated by WRKY33 upon submergence treatment.

**Conclusions:**

Our study has identified a new putative binding motif of WRKY33 and recovered numerous previously unknown target genes of WRKY33 during submergence stress. The *WRKY33* gene positively participates in flooding response probably by transcriptional regulation of the downstream submergence-related target genes via a “TC box”.

**Supplementary Information:**

The online version contains supplementary material available at 10.1186/s12863-021-00972-5.

## Background

Large areas of cropland in the world are subject to seasonal flooding, which causes significant losses to crop production almost every year. The diffusion of oxygen in water is 10,000 times slower than that in air [1], drastically reducing the supply of oxygen to the plants. Morphological adaptations of plants to low-oxygen stress include the formation of adventitious roots, as well as the development of cortical air spaces in roots that promote air transport [2]. Meanwhile, the induction of fermentation pathway enzymes has been established as an important metabolic adaptation to anaerobiosis [3, 4]. Over the last decade, it has become increasingly evident that the N-degron pathway plays a well-characterized role in the response to hypoxia through flooding and plant submergence [5, 6]. In addition, a variety of transcription factors (TFs) have been reported to regulate gene expression that promotes adaptive responses to the environmental and physiological stress [7], including the *Dof* (DNA-binding with one finger) gene family [8], the MADS-box gene family [9], and the *WRKY* gene family.

The WRKY TF family, found exclusively in green plants, is characterized by the highly conserved amino acid sequence WRKYGQK at the N-terminus and the zinc-finger structure at the C-terminus [10]. Numerous studies have demonstrated that WRKY TFs are involved in regulation of various processes, such as seed germination, leaf senescence, and the responses to biotic and abiotic stresses [11, 12]. In particular, one member of the WRKY TF family, WRKY33, has been shown to regulate plant defense responses to a variety of stresses [13, 14]. For example, previous studies have documented that overexpression of the *WRKY33* gene enhances the resistance to oxidative stress [15] and promotes pathogen defense [16]. In addition, our recent study found that overexpression of *WRKY33* can enhance the submergence tolerance of *Arabidopsis* mainly via directly up-regulating the gene *RAP2.2* [17]. We further revealed that WRKY33 together with WRKY12 in up-regulating *RAP2.2* expression during submergence response, meanwhile *WRKY33* level is increased in *RAP2.2*-overexpressing plants and further experiments confirmed a positive feedback regulation of *WRKY33* by RAP2.2 during submergence response in *Arabidopsis thaliana* [17]. It has been shown that WRKY33 acts as a key factor in submergence response of *Arabidopsis thaliana*, however the downstream regulatory network governed by WRKY33 is still poorly understood. In this work, we used ChIP-seq to identify all WRKY33-targeted genes in response to submergence, which will provide a more clearly regulation pathway mediated by WRKY33.

## Results

### Verification of the function and phenotype of *35S:FLAG-WRKY33* transgenic *Arabidopsis* in submergence response

A previous study showed that *WRKY33* was induced by hypoxia stress in roots of *Arabidopsis* [4]. Recently, *WRKY33* was reported to positively regulate submergence response via interacting with WRKY12 to directly upregulate *RAP2.2* in *Arabidopsis* [17]. To further identify other WRKY33 targeted genes during submergence response at a genome-wide scale, we use *35S:FLAG-WRKY33* overexpression transgenic plants (*WRKY33-OE*) upon 24 h’ submergence treatment for ChIP-seq. Before the ChIP experiment, we obtained the *WRKY33OE* transgenic plants (Supplemental Fig. 1) in Col background and examined its submergence tolerance to make sure that the plants were workable. The phenotypic assay showed that *WRKY33OE* plants were more tolerant to submergence treatment compared to Col (Supplemental Fig. 2A). Survival rates and dry weights of Col, *WRKY33OE-1* and *WRKY33OE-2* plants were also consistent with their phenotypic assays (Supplemental Fig. 2B-C). Malondialdehyde (MDA) contents (Supplemental Fig. 2D) were also evaluated among Col, *WRKY33OE-1* and *WRKY33OE-2* plants and the results also supported that overexpression of *FLAG-WRKY33* enhanced the submergence tolerance in *Arabidopsis*. Compared to wild-type, the results indicate that *WRKY33-OE* transgenic plants could be used to identify downstream targets of WRKY33 via ChIP-seq.

### Analysis of the ChIP-seq peaks

**Fig. 1:**
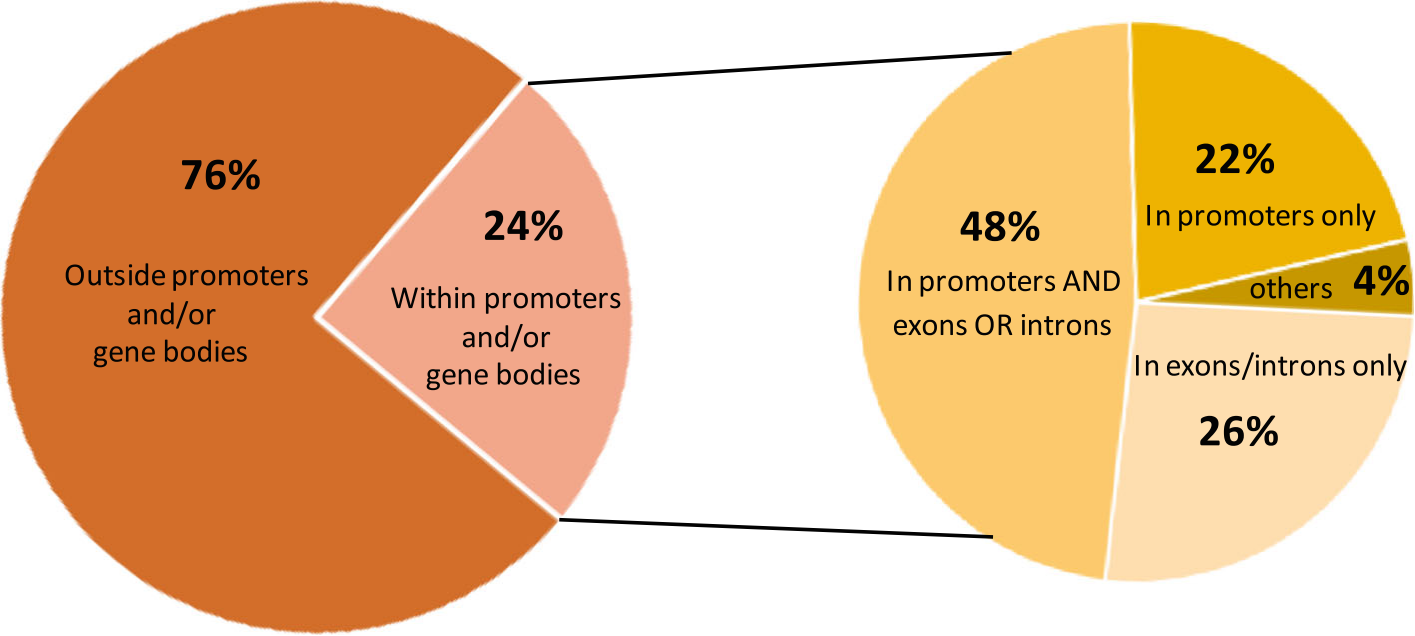
Distribution of ChIP peaks in the genome. Percentage of peaks that reside 2 kb upstream of the transcription start site or 2 kb downstream of the stop codon (gene body), and location of the peaks in the gene bodies

**Fig. 2:**
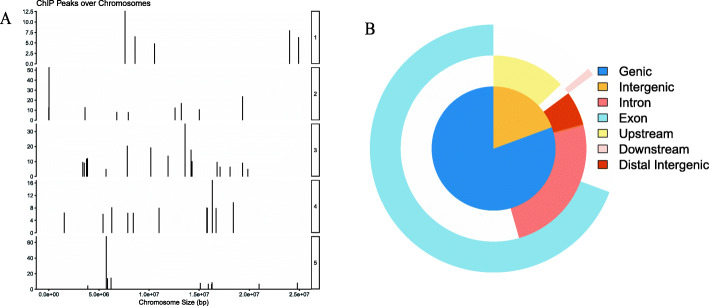
The location of all ChIP peaks over chromosome. **a** ChIP peaks coverage plot: the right ordinate represents the chromosome, the left ordinate represents the size of the peak, and the abscissa represents the size of the chromosome. **b** Genomic Annotation by vennpie. Visually shows the full annotation with their overlap

Having confirmed that the *WRKY33OE* transgenic plants had the enhanced submergence resistance, we then performed the ChIP experiment firstly by using the samples (2 g pooled leaf materials) of 14-day-old seedlings of *WRKY33OE1* and *WRKY33OE2* plants after submergence treatment for 24 h. The average size of the input fragments and the anti-FLAG ChIP libraries were approximately 100–400 bp. The immunoprecipitated DNA fragments were then sent to the BGI (Shenzhen, China) company for further sequencing. The input library had 25.4 million reads and the FLAG Ab ChIP library had 24.6 million reads. More than 95% of the reads were mapped to the *Arabidopsis* genome. The MACS2 program (Analysis based on ChIP-seq models) [18] was used to identify the enriched regions using a false discovery cutoff of 0.05. The location of the enriched peaks in the *Arabidopsis* genome is shown in the supplemental Table 1 (Additional file 3). Of the 393 enriched regions, 24% of the peaks were in genetic regions (from 2 kb upstream of the start of transcription to 2 kb downstream of the stop codon, including the coding region). Of the peaks that were in the genetic regions, 22% located only in the promoter regions, 48% in the promoter and exons or introns regions, only 26% in exons and introns (Fig. 1). After calling peak, we aimed to examine the peak locations among the whole genome. We then used the covplot function in ChIPseeker (an R package for ChIP peak Annotation, Comparison and Visualization) to calculate the coverage of peak regions over the chromosomes. We generated a figure for visualization (Fig. 2a). Since some annotations overlapped, we then viewed the complete annotations with overlap through the vennpie function in ChIPseeker (Fig. 2b). Table 1 lists the genes related to the peaks in the gene region. These peaks are enriched by more than 5-fold and all have known putative functions.

**Table 1:** List of genes and their putative function

Gene Name	Putative Function	Fold-Change
AT1G21650	Preprotein translocase SecA family protein	8.0
AT1G64628	conserved peptide upstream open reading frame 57	5.2
AT2G01008	maternal effect embryo arrest protein	8.1
AT2G15540	non-LTR retrotransposon family	5.2
AT2G18220	Noc2p family	5.2
AT2G29350	senescence-associated gene 13	7.7
AT2G31040	Encodes an integral thylakoid protein that facilitates assembly of the membranous part of the chloroplast ATPase	9.6
AT2G47090	zinc ion binding/nucleic acid binding protein	12.4
AT3G10810	zinc finger (C3HC4-type RING finger) family protein	6.4
AT3G11280	Duplicated homeodomain-like superfamily protein	5.9
AT3G11900	aromatic and neutral transporter 1	7.1
AT3G12120	fatty acid desaturase 2	7.8
AT3G22160	JAV1 is a repressor of jasmonate-mediated defense responses	11.3
AT3G22170	far-red elongated hypocotyls 3	9.5
AT3G27503	Encodes a member of a family of small, secreted, cysteine rich proteins with sequence similarity to SCR	10.6
AT3G30250	transposable element gene	8.0
AT3G33058	gypsy-like retrotransposon family	15.7
AT3G41768	rRNA	10.2
AT3G41979	5.8SrRNA	6.8
AT3G42130	glycine-rich protein	6.2
AT3G45755	transposable element gene	6.1
AT3G52140	tetratricopeptide repeat (TPR)-containing protein	6.2
AT4G10030	Alpha/beta hydrolase domain containing protein involved in lipid biosynthesis	5.3
AT4G20360	Nuclear transcribed, plastid localized EF-Tu translation elongation factor	5.2
AT4G32700	helicases;ATP-dependent helicases;nucleic acid binding;ATP binding;DNA-directed DNA polymerases;DNA binding	5.3
AT4G32810	carotenoid cleavage dioxygenase 8	5.2
AT4G34035	pre-tRNA tRNA-Arg	9.6
AT4G34040	RING/U-box superfamily protein	7.9
AT4G35090	catalase 2	5.2
AT4G39672	pre-tRNA	6.1
AT5G17420	Encodes a xylem-specific cellulose synthase that is phosphorylated on one or more serine residues	30.1
AT5G17730	P-loop containing nucleoside triphosphate hydrolases superfamily protein	8.0
AT5G18650	CHY-type/CTCHY-type/RING-type Zinc finger protein	8.5
AT5G37960	GroES-like family protein	5.4
AT5G40690	histone-lysine N-methyltransferase trithorax-like protein	6.1
AT5G61710	cotton fiber protein	5.3

The genes listed in this table are limited to those associated with peaks that were enriched greater than 5-fold and have been classified with a known function

### Motif analysis of WKRY33 TF targeted genes

**Fig. 3:**
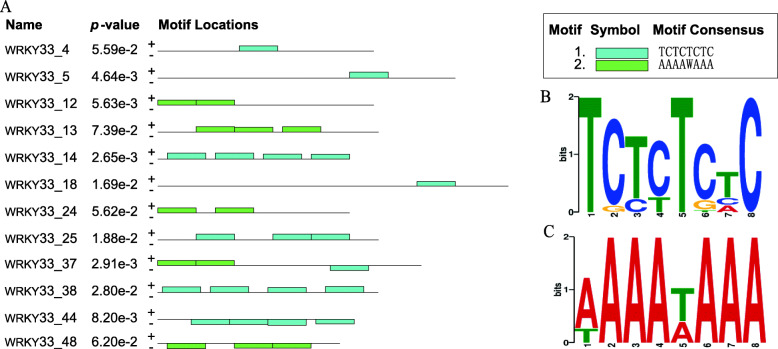
Genome-wide distribution of WRKY33 binding sites in the A*rabidops*is genome identified by ChIP-seq. **a** MEME-CHIP analysis of *WRKY33* motif. *Arabidopsis* reference genome (TAIR10) by Bowtie, Among the two motifs identified by MEME, ChIP peaks and *p* value and locations where two motifs are located. **b-c** The two most representative motif patterns

We analyzed all the promoter-located peak sequences from the ChIP-seq using MEME-ChIP [19] to identify the enriched motif, and detected the two types of motifs (Fig. 3a). The most significantly enriched MEME motif is “TCTCTCTC” (E-value of 6.3e-005) which is different from the “W box” bound by WRKY33 TF reported previously. We then named it as “TC box” (Fig. 3b). The next most significant motif is AAAAWAAA (E-value of 3.1e+ 002) (Fig. 3c). WRKY proteins can repress or activate the expression of downstream genes via binding to the W-box (TGACC (A/T)) in promoter of its target genes upon pathogen defense [18]. The identified “TC box” motif may responsible for the activation or repression of submergence-related target genes which still needs further verifications.

### Gene ontology analysis to identify biological and functional enriched categories

**Fig. 4:**
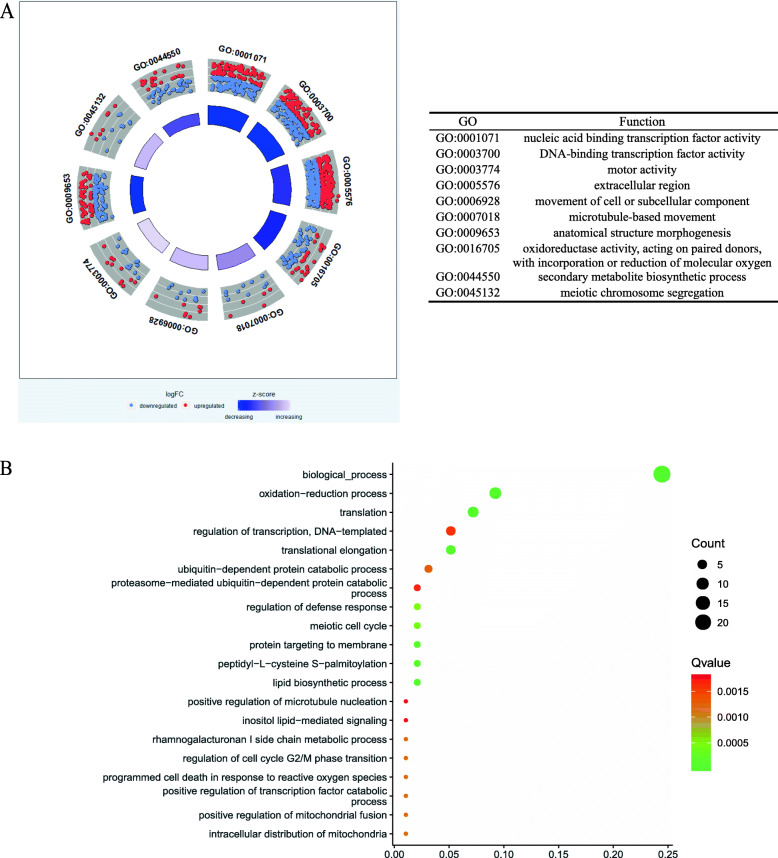
**a** Top 10 significantly enriched GO biological processes of WRKY33BGSs. Red and blue dots indicate up-regulated DEGs and down-regulated DEGs enriched in the term respectively, and a z-score indicated in the inner quadrangle. **b** The results of the top 20 extremely significant enrichments indicate that the gene ontology categories for biological processes includes the oxidation-reduction process and programmed cell death in response to reactive oxygen species

Gene Ontology (GO) analyses using the Enrich GO [20] revealed 61 GO categories belonging to the Biological Process (BP) ontology, which were determined to be significantly over-represented in the ChIP-seq sample relative to the *Arabidopsis* genome (fisher < 0.01, Additional file 4). The top 10 significantly enriched GO biological processes of WRKY33BGSs were shown in Fig. 4a. The results of the top 20 extremely significant enrichments (Fig. 4b) suggest that the gene ontology related to the submergence response includes the oxidation-reduction process, programmed cell death in response to reactive oxygen species and lipid biosynthesis process. Additional biological processes including cellular response to auxin stimulus, response to hydrogen peroxide were also identified when using a fisher greater than 0.01 and less than 0.05 (Additional file 4). Plant phytohormones, such as auxin, may also participate in the submergence response process as suggested by our Gene Ontology (GO) analysis, which still needs further experimental validation.

### Expression analysis of genes contain the “TC box” in Col and *WRKY33OE* plants after submergence treatment

**Fig. 5:**
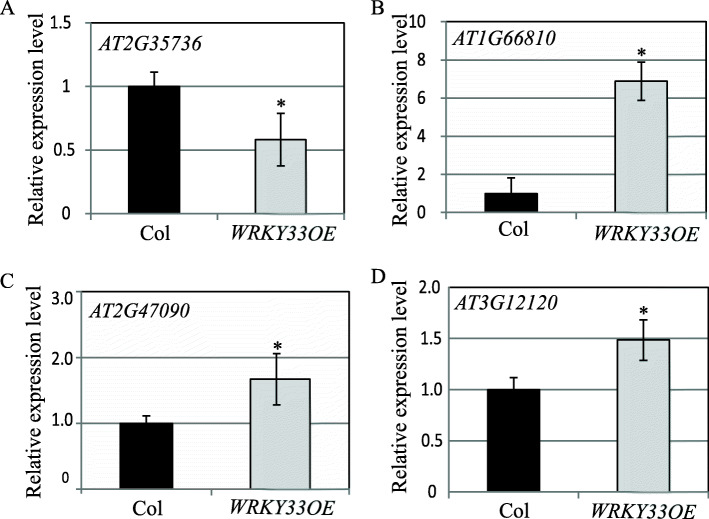
Expression analysis of genes containing the “TC box” in Col and *WRKY33OE* plants after submergence treatment. **a** *AT2G35736* gene is downregulated by WRKY33 upon submergence treatment for 24 h. **b-d** *AT1G66810*, *AT2G47090* and *AT3G12120* genes are upregulated by WRKY33 upon submergence treatment for 24 h. Three independent biological replicates were used. Data are average values ±SD (*n* = 3) of 3 biological replicates. *(*p* < 0.05, according to Student’s *t*-test) indicates significant difference from Col

WRKY33 may regulate its downstream target genes directly via the identified “TC box” during submergence response. To further validate this hypothesis, we selected four genes that contain the “TC box” and performed a qPCR test. The results showed the expression levels of these four genes were all regulated by WRKY33 transcription factor. *At2G35736* gene was downregulated by WRKY33 while the other three genes *At1G66810*, *At2G47090*, and *At3g12120* were upregulated by WRKY33 (Fig. 5). These results support that these four genes targeted by WRKY33 may participate in submergence response via the “TC box”. However, further experimental validations including EMSA (electrophoretic mobility shift assay) are needed in the future to fully validate the direct regulation role of WRKY33.

## Discussion

Flooding stress, one of the most important abiotic stresses, has attracted the attention of scientists over the world [21]. Many studies have revealed the molecular mechanisms of plants in response to flooding [21]. A few genes from the WRKY transcription factor family have been shown to play an important role in submergence response, including, WRKY22 [22] and WRKY33 [17]. WRKY22-mediated pathways in response to submergence have shown to regulate multiple transcription factors, including WRKY29 and WRKY53 [22]. The WRKY33/WRKY12-*RAP2.2* feedforward cycle in submergence response we discovered recently has confirmed the key positive role of WRKY33 in flooding response [17]. In this work, we went a step further and tried to explore the regulation network of WRKY33 during submergence stress. By phenotypic analysis, we found that plants overexpressing *FLAG-WRKY33* did enhance the resistance to submergence stress compared with Col (Supplemental Fig. 2). We then used *35S:FLAG-WRKY33* overexpressing transgenic lines (*WRKY33-OE*) upon submergence treatment for ChIP-seq, to identify the WRKY33 TF target genes at a genome-wide scale. By ChIP-seq analyses, we identified 104 WRKY33-binding genes upon submergence stress (WRKY33BGSs) and gene enrichment analysis showed that these genes participate in oxidoreductase reactions, lipid biosynthetic process and other functions. Most of these identified genes are reported for the first time for submergence stress. The major motif that we identified in the WRKY33BGSs promoters is the “TC box” cis-element. This candidate motif for WRKY33 TF may regulate genes expression during submergence stress. Our further functional analyses of all identified genes suggest that WRKY33BGSs may protect cells from oxidative stress and other processes to improve the tolerance ability upon submergence stress.

The identified “TC box” cis-element is a new motif different from the known “W box” element for WRKY33 and may be specific to regulating the target genes during submergence stress. WRKY33 can regulate *RAP2.2* expression via the W box element only during the submergence response [17]. Interestingly, there also is a “TC box” sequence “TCTCTC” in the promoter region (− 1, 875 bp) of *RAP2.2*. Previous studies have shown that the TFs have different binding abilities towards different cis-elements upon different conditions. For example, IPA1 was reported to bind to the “GTAC” element in the promoter of *DEP1* in the normal condition while bind to the “TGGGCC” element in the promoter of *WRKY45* upon pathogen infection [23]. This switch is mediated by the phosphorylation of IPA1 protein. Submergence treatment might also induce the phosphorylation of WRKY33 like IPA1 upon pathogen infection [17, 21]. In addition, this TF may also have different binding abilities towards “W box” or “TC box” elements between normal growth and submergence treatment conditions like IPA1. Such a difference in binding ability may be mediated by the protein post-transcriptional modifications of WRKY33.

In this study, we obtained a more comprehensive understanding of the submergence stress response mediated by WRKY33. The ChIP-seq candidate genes regulated by WRKY33 provide a more comprehensive understanding of the molecular basis of plant submergence response. These genes can be further manipulated to improve stress tolerances when their functions and regulation pathways are well clarified. In addition, the functions of genes induced by low-oxygen stress seem to overlap those induced by other biotic or abiotic stress responses [24]. It is worth noting that only roles of WRKY33 in leaves during submergence response were examined here. However, its function may be altered by using different tissues, since *WRKY33* also is highly expressed in roots [25]. The hypoxic response including many physiology processes, such as aerobic metabolism, carbon and energy partition, redox balance, ethylene accumulation, gene regulation cascades [26] and so on, is complex. The work we have done is just the tip of the iceberg and more works are still needed to clarify the mechanism of submergence response of plants in the future.

## Conclusion

We identified numerous previously unknown direct target genes of WRKY33 in response to submergence stress by ChIP-Seq and a new cis-element “TC box” was identified. Our work suggested that WRKY33 TF may positively participates in flooding response via the “TC box” to its target genes. Thus, our results provide new insights into the functions of WRKY33 transcription factor and the submergence response of *Arabidopsis*.

## Methods

### Arabidopsis growing conditions and submergence treatment

Briefly, cDNA was prepared from 4-week rosette leaves of *Arabidopsis* and was diluted to 50 times. The diluted cDNA was then used as a template to amplify the *WRKY33*, which was inserted into a vector tagged by FLAG tag, under the control of the *35S* promoter. The construct was transformed into *Agrobacterium* strain GV3101 [27], which was used to transform *Arabidopsis* using the floral dip method and identified by hygromycin screening followed by qRT-PCR analysis of their expression levels. The *35S:FLAG-WRKY33* (*WRKY33OE*) transgenic plants we used were obtained in this work. All materials were grown at 22 °C in a 16-h light/8-h dark cycle. Seeds were germinated on 1/2 MS medium (pH = 5.85) for 7 days and then transplanted into soil.

For submergence treatments, 4-week-old plants were submerged 10 cm below the surface of the water in darkness for 50 h. All submergence treatments started at 9:00 a.m. Twelve Col and *WRKY33OE* plants were used for submergence treatment every time. The total experiments were repeated three times.

For ChIP-sequencing, 4-week-old *35S:FLAG-WRKY33* transgenic plants were submerged 10 cm below the surface of the water in darkness for 24 h. Then rosette leaves were collected for ChIP experiments. All submergence treatments started at 9:00 a.m.

### Malondialdehyde measurements

The Malondialdehyde (MDA) was measured according to a previous study [28]. 4-week-old rosette leaves of 10 plants treated by dark submergence were weighed and pulverized in 5% trichloroacetic acid buffer, and then mix the supernatant with 6.7% thiobarbituric acid and 5% trichloroacetic acid buffer. The materials were further incubated at 100 °C for 0.5 h, and then cooled to the room temperature. The absorbance was measured at 532, 450, and 600 nm with a spectrophotometer plate reader.

### ChIP and ChIP-sequencing

Samples of 14-day-old seedlings of *WRKY33OE1* and *WRKY33OE2* plants were dark submergence treated for 24 h and fixed using 1% formaldehyde and prepared for chromatin immunoprecipitation assays, as previously described [29]. The DNA-protein complexes were extracted from rosette leaves (2 g pooled leaf materials) of 4-week-old *35S:FLAG-WRKY33 OE1* and *OE2* transgenic plants, and pulled down using anti:FLAG antibody (Sigma-Aldrich F1084) and protein A Agarose beads following the ChIP protocol [30]. The immunoprecipitated DNA fragments were dissolved in 40 μl ddH2O and then sent to the BGI (Shenzhen, China) company for the following experiment. 10% of the total DNA-protein complexes before the immunoprecipitation were used as the input DNA.

ChIP-seq service was performed by BGI company (Shenzhen, China). The DNA is combined with End Repair Mix and incubated at 20 °C for 30 min. We further purified the end-repaired DNA with QIAquick PCR Purification Kit (Qiagen), and added A-Tailing Mix and incubated at 37 °C for 30 min. We combined the purified Adenylate 3 ‘Ends DNA, Adapter and Ligation Mix and incubated the ligation reaction at 20 °C for 15 min. We purified the Adapter-ligated DNA with the QIAquick PCR Purification Kit. We conducted several rounds of PCR amplification with PCR Primer Cocktail and PCR Master Mix to enrich the Adapter-ligated DNA fragments. Then the PCR products are selected (about 100–300 bp, including adaptor sequence) by running a 2% agarose gel to recover the target fragments. We purified the gel with QIAquick Gel Extraction kit (QIAGEN). The final library was quantitated in two ways: determining the average molecule length and sample integrity and purity using the Agilent 2100 bioanalyzer instrument (Agilent DNA 1000 Reagents) and quantifying the library by real-time quantitative PCR (qPCR). The double stranded PCR products were heat-denatured and circularized by the splint oligo sequence. The single strand circle DNA (ssCir DNA) was formatted as the final library. Library was qualified by Qubit ssDNA kit. The sequencing was performed with the BGISEQ-500 sequencing system, featured by combinatorial probe-anchor synthesis (cPAS) and DNA Nanoballs (DNB) technology for superior data quality (BGI-Shenzhen, China).

The raw sequencing image data were examined by the Illumina analysis pipeline. ChIP-seq reads were aligned to the *Arabidopsis* reference genome (TAIR10) by Bowtie [31] with at most 2 mismatches. The input group was used as a control. The results were visualized with IGV software. Reads that appeared more than twice at the same position on the same strand were discarded to remove PCR duplication. MACS2 (Model-based Analysis of ChIP-seq) [32] was used to identify peaks using a q-value cutoff of 0.05.

### Motif analysis

To identify possible binding motif of the WRKY33 transcription factor, the ChIP peak sequences were subjected to MEME (Multiple EM for Motif Elicitation)-ChIP [19]. The MEME-ChIP program uses two ab initio motif discovery algorithms: MEME [19], and DREME (Discriminative Regular Expression Motif Elicitation) [33], which uses regular expressions to search for short eukaryotic TF motifs that are missed by MEME.

### Gene function of WRKY33 TF target genes

In order to determine the putative functions of the target gene *WRKY33*, all identifed genes with ChIP-seq peaks in the upstream promoter region or the potential regulatory region downstream were subjected to annotation of the categories of ontological genes (GO) [20]. The default Fisher’s Exact Test and Benjamini-Yekutieli multiple test correction methods [34] were used to generate *p*-values for statistical significance and corresponding False Discovery Rate (FDR) values.

### RNA extraction and quantification

Total RNA was isolated using the Biospin Plant Total RNA Extraction kit according to the user manual (Bioer Technology; Hangzhou, China), from the pooled three-week old rosette leaves of Col and *35S:FLAG-WRKY33* plants, and 1–2 μg total RNA was used for reverse transcription, using the PrimeScript RT reagent kit (Takara Cat# RR047A). A QuantiNova SYBR Green PCR Kit was used for qPCR reactions with qPCR-specific primers. The expression levels of putative target genes were compared with *Arabidopsis ACTIN* genes.

## Supplementary Information

**Additional file 1: Supplemental Fig. 1.** Identification of WRKY33 overexpressing transgenic plants.

**Additional file 2: Supplemental Fig. 2.** WRKY33 positively regulates the submergence response in Arabidopsis.

**Additional file 3.** List of enriched peaks and their location in the Arabidopsis genome.

**Additional file 4.** The putative function of the target gene WRKY33. And primers used in this study.

**Additional file 5.** Primers used in this study. Primers used for vector construction and gene expression analysis.

## Data Availability

All data generated are included in this published article and its supplementary files. The raw sequence data reported in this paper have been deposited in the Genome Sequence Archive (Genomics, Proteomics & Bioinformatics 2017) in National Genomics Data Center (Nucleic Acids Res 2021), China National Center for Bioinformation / Beijing Institute of Genomics, Chinese Academy of Sciences, under accession number CRA003775 that are publicly accessible at https://bigd.big.ac.cn/gsa.

## Abbreviations

At: *Arabidopsis thaliana*
ChIP: Chromatin immunoprecipitation
DREME: Discriminative Regular Expression Motif Elicitation
GO: Gene Ontology
MEME: Multiple EM for Motif Elicitation
RT: Reverse transcriptase
seq: Sequencing
TF: Transcription factor
