# Supplementary material for: Genome-wide (ChIP-seq) identification of target genes regulated by WRKY33 during submergence stress in Arabidopsis
Source: BMC Genom Data. 2021 May 24;22:16. doi: 10.1186/s12863-021-00972-5 (PMC8142642; doi:10.1186/s12863-021-00972-5)
Supplement: Supplementary file 1 — Additional file 1: Supplemental Fig. 1. Identification of WRKY33 overexpressing transgenic plants. [file 12863_2021_972_MOESM1_ESM.docx]

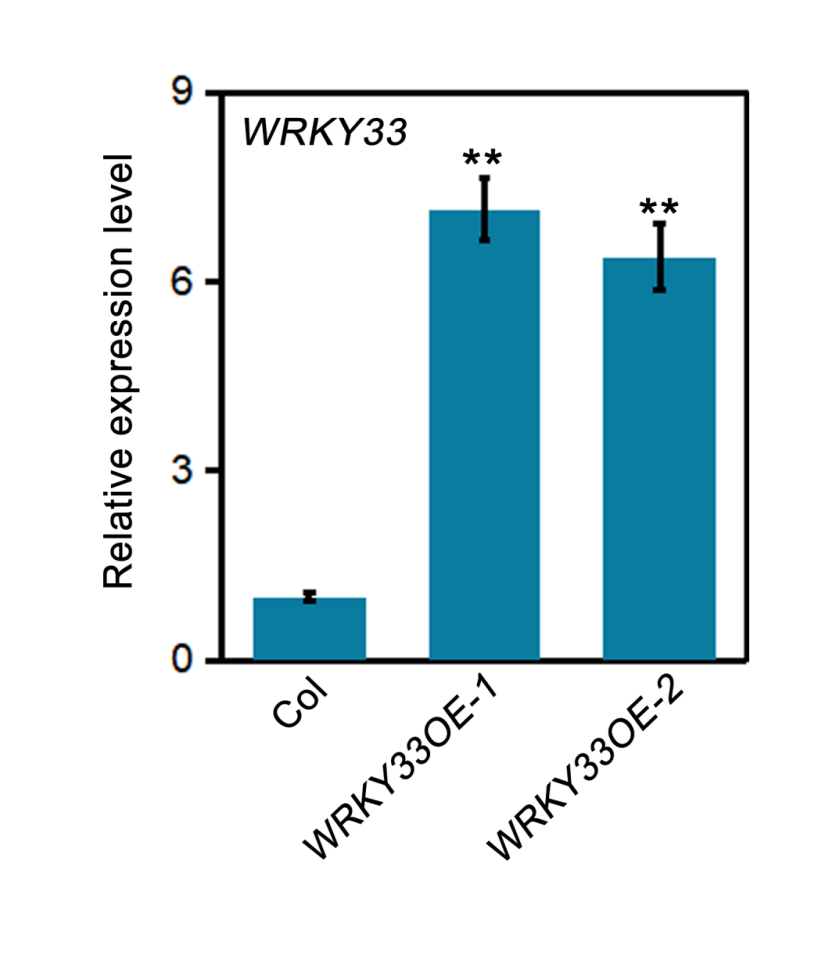


**Supplemental Figure 1.** Identification of *WRKY33* overexpressing transgenic plants.

Total RNA was extracted from 4-week-old rosette leaves of Col and *WRKY33OE* plants line1 and line2. ** (p < 0.01, according to Student’s *t*-test) indicates significant difference from Col.
