# Supplementary material for: Genome-wide (ChIP-seq) identification of target genes regulated by WRKY33 during submergence stress in Arabidopsis
Source: BMC Genom Data. 2021 May 24;22:16. doi: 10.1186/s12863-021-00972-5 (PMC8142642; doi:10.1186/s12863-021-00972-5)
Supplement: Supplementary file 2 — Additional file 2: Supplemental Fig. 2. WRKY33 positively regulates the submergence response in Arabidopsis. [file 12863_2021_972_MOESM2_ESM.docx]

**
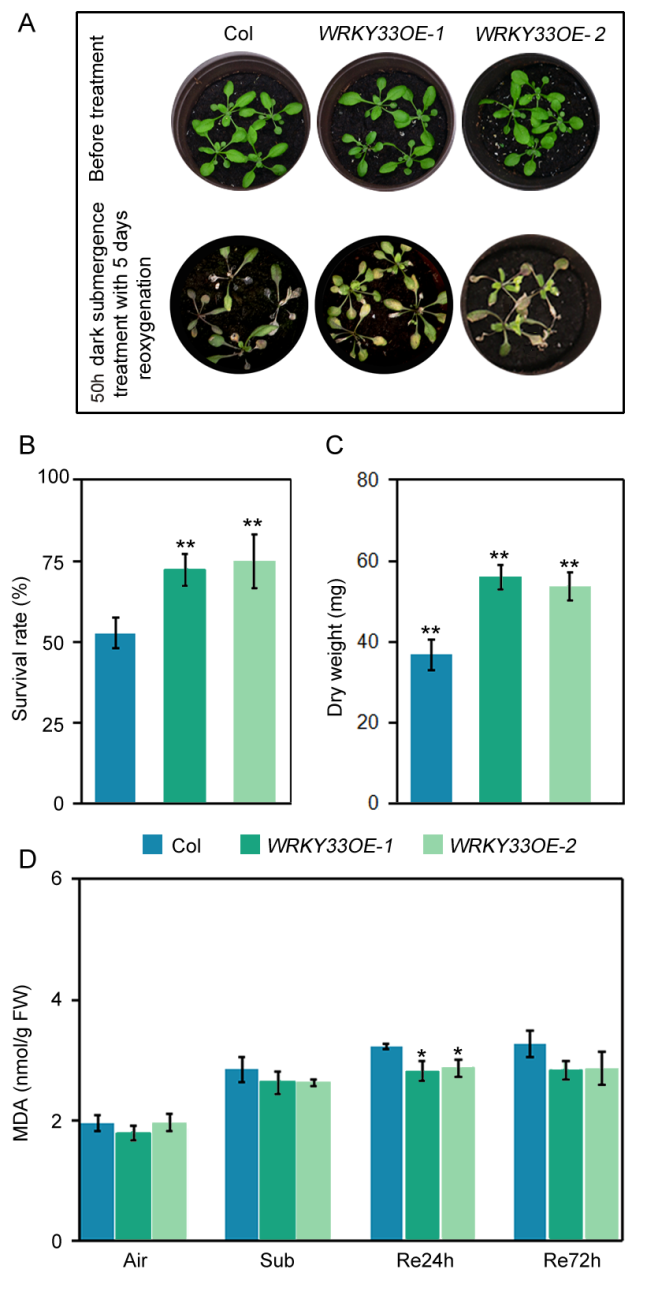
**

**Supplemental Fig. 2** *WRKY33* positively regulates the submergence response in *Arabidopsis*.

(A) Phenotypes of Col, *WRKY33OE-1/2* plants in response to submergence. (B) Survival rates of Col, *WRKY33OE-1/2* plants after 50h submergence treatment and 5 days recovery. (C) Dry weights of Col, *WRKY33OE-1/2* plants after 50h submergence treatment and 5 days recovery and drying for 2 days. (D) Malondialdehyde content of Col, *WRKY33OE-1/2* plants, (Air) before submergence, (Sub) after 2 days of submergence, (Re24 h) subsequent recovery for 24 h or (Re72 h) 72 h. FW: fresh weight. **(p < 0.01, according to Student’s *t*-test) and *(p < 0.05, according to Student’s *t*-test) mean significant differences between values.
